# Supplementary material for: Hepatitis B infection: Evaluation of demographics and treatment of chronic hepatitis B infection in Northern-western Tanzania
Source: PLoS One. 2024 Oct 8;19(10):e0309314. doi: 10.1371/journal.pone.0309314 (PMC11460692; doi:10.1371/journal.pone.0309314)
Supplement: Attachment — Submitted filename: Response to reviewers.docx [file pone.0309314.s001.docx]

**Reviewers’ responses**

| **Requirements/comments** | **Response to the requirement/comment** | **Location** |
| --- | --- | --- |
| **Journal Requirements** |  |  |
| 1. Manuscript meets PLOS ONE's style requirements, including those for file naming | Responded accordingly | Elsewhere in the manuscript |
| 2. Concerning participants’ consent | Responded accordingly | Page 2,9 |
| 3. Delete this sentence: Furthermore, the patients were categorized into male and female groups based on sex | Sentence deleted accordingly | Page 13 |
| 4. **Sex** should be replaced with gender throughout the manuscript and tables | Responded accordingly | Page 15 and elsewhere in the manuscript |
| 5. **HBVL** should be replaced with HBV DNA. | Responded accordingly | Elsewhere in the manuscript |
| 6. The HBV DNA cutoff of **20000** should be replaced with 20,000 throughout the manuscript and tables. | Responded accordingly | Elsewhere in the manuscript |
| 7. Many complicated sentence structures should be re-written for clarity.  For example,   7a. Among the efforts to prevent HBV infection and therefore reduce the risk of developing chronic HBV infection, in 1992, the WHO summoned every country to integrate HBV vaccination into their universal childhood vaccination programs by 1997 [7] so as to reduce the risks of acquiring HBV infection and therefore acute hepatitis B which can lead to chronic hepatitis B infection. | Responded accordingly | Page 3 |
| 7b.  Our results have shown that, although nearly all chronic hepatitis B-infected patients were older than 20 years, which was probably due to the effect of the national infantile-pediatric HBV vaccination program that started about 20 years ago, anti-HBV antiviral therapy uptake among the eligible patients according to the 2015 WHO-HBV prevention, management, and care guidelines for HBV infection was remarkably lower than the expectation of the WHO towards the elimination of HBV by 2030. | Rephrased accordingly | Page 20 |
| This statement implies that HBV treatment rollout was not country-wide. Is that indeed the case?  ·      Tanzania introduced HBV treatment in 2009 at the Bugando Medical Centre and in 2016 at Muhimbili National Hospital [8]. | YES. Readdressed more informatively. | Page 4, 5 |
| Batch testing for HBV DNA is mentioned in the Methods.  However, whether positive pools were then de-complexed and tested individually is not stated.  This is essential for accurate determination of HBV DNA prevalence and levels. | The test done is quantitative real-time PCR and not qualitative. All participants were known HBV positive patients attending at Bugando Medical Centre-HBV clinic. The results were either target not detected (TND) which would be treated as negative in qualitative PCR. TND was treated as zero value. Other results were <20IU/mL, 20IU/mL to 1.7 X10^8^IU/mL or >1.7 X10^8^IU/mL.  It well explained accordingly | Page 11 |
| Study limitations – there are several – should be clearly stated in the Discussion.  The study population is modest in size. | Study limitations revised and discussed accordingly | Page 26, 27 |
| The Discussion is quite long and should be shortened / streamlined. | The discus section sectioned revised and shortened | Page 20-27 |
| **Review Comments to the Author** |  |  |
| **Reviewer #1:** |  |  |
| 1. Title: very long. can be shortened and made more explicit | Title shortened and improved accordingly | Page 1 |
| 2. Methods: The methods section combines a chart review with newly recruited patients. This creates a problem as the methods section technically must be reproducible. There is no way of knowing if the same laboratory methods, under similar conditions was used to generate data collected from the chart review. The study can be salvaged by describing either of the groups independently as two studies and not combining their data. Data Analysis, Discussion and Conclusion: Due to the anomaly in the methods section, the data analysis, discussion and conclusion are not valid. | The prospective and retrospective participants described separately | Page 10-13 |
| **Reviewer #2:** |  |  |
| Introduction |  |  |
| 1. The general situation of HBV treatment in Tanzania needs to be clarified. Are Bugando Medical Center and Muhimbili National Hospital the only sites that treat HBV in Tanzania? | YES. Currently HBV treatment is available at Bugando Medical centre and Muhimbili National Hospital. The statement re-written to reflect the concept. | Page 4, 5 |
| 2. Is there any information on the number of individuals on treatment among those diagnosed with HBV in Tanzania? | Data on HBV treatment is scarce in Tanzania. The MNH project data have not yet published. Negligible information is available from Bugando Medical Centre. | Page 4 |
| Methodology |  |  |
| 3. I suggest that you briefly explain the principle of the formula used to calculate the sample size, including the numbers. | Responded accordingly | Page 9, 10 |
| 4. Is there information in the database about testing for other pathogens such as HIV, HCV, and HDV? Is testing routine in Tanzania? | HIV and HCV are routinely done in Tanzania, however in the prospective patients, these tests were not done. Also in the retrospective patients, these data were not available | Page 11, 12 |
| 5. Is there data on the algorithm used for HBsAg testing in the patients included in the study? Which test is used? | There is HBsAg testing algorithm in Tanzania. Tanzania follows the 2015 WHO guidelines for HBsAg testing, treatment and monitoring of HBV management | Page 5, 6 |
| Results |  |  |
| 6. Tables 1 and 2 could be merged into a single table. This would give a better view of the results. | Table 1 and 2 combined (now table but with three columns) | Page 15, 16,17 |
| 7. Tables 1 and 2 can have two columns. One with the characteristic and the other with the absolute number and percentage, just indicate it in the column legend. | Table 1 and 2 combined (now table but with three columns) | Page 15, 16, 17 |
| 8. Other factors such as co-infection with HBV, HDV, and HCV were not taken into account in the eligibility for treatment. If there is no data, it is important to discuss it. | Coinfection of HBV with HIV, HDV and HCV were not available on routinely testing as explained in the manuscript. These limitations are well discussed | Page 11, 12, 26, 27 |
| Discussion |  |  |
| 9. The WHO recommendations for treatment were updated in 2024. The study was carried out under the 2015 recommendations, but it is also important that the results are discussed in the context of the new recommendations. | It was considered accordingly | Page 4, 6, 7, 17, 19 |
